# 2170. It takes two to tango: ß-lactam/ß-lactamase inhibitor combinations targeting metallo-ß-lactamase (MBL)- producing *Klebsiella pneumoniae.*

**DOI:** 10.1093/ofid/ofad500.1792

**Published:** 2023-11-27

**Authors:** Jan Naseer Kaur, Jack F Klem, Navaldeep Singh, Patricia N Holden, Raymond Cha, Liang Chen, Barry N Kreiswirth, Nicholas M Smith, Brian T Tsuji

**Affiliations:** University at Buffalo, Buffalo, New York; University at Buffalo, Buffalo, New York; University at Buffalo, Buffalo, New York; University at Buffalo, School of Pharmacy & Pharmaceutical Sciences, Buffalo, New York; Univ at Buffalo SUNY School of Pharmacy, Buffalo, New York; HMH-CDI, Nutley, New Jersey; Center for Discovery and Innovation, Hakensack Meridian Health, Nutley, New Jersey; University at Buffalo, School of Pharmacy & Pharmaceutical Sciences, Buffalo, New York; University at Buffalo, School of Pharmacy & Pharmaceutical Sciences, Buffalo, New York

## Abstract

**Background:**

The emergence of pan drug resistant (PDR) gram-negatives co-producing NDM-1 and CTX-M-15 calls for devising more efficient biocidal regimens. Also, understanding the salient transcriptomic and phenotypic changes that contribute to persistence which aid in pathogen survival is critical to therapeutic preparation in the clinical setting.

**Methods:**

A 10-day hollow fiber infection model (HFIM) was utilized to simulate humanized β-lactam regimens involving imipenem (IMI) 1g q8h, aztreonam (ATM) 2g q8h, and ceftazidime/avibactam (CAZ/AVI) 2g/500mg alone and in 3-drug combination regimens against a PDR *Klebsiella pneumonia* isolate co-harboring bla*_NDM-1_* and bla*_CMY-6_*, bla_CTX-M-15_ and bla*_SHV-2_*. Static time kills were performed to investigate the interplay between IMI and ATM. At 2h post drug exposure, total RNA was analyzed by qRT-PCR for the expression of pertinent genes. Glutaraldehyde-fixed samples were imaged using fluorescence and scanning electron microscopy.

**Results:**

In HFIM, IMI contributed synergistically to the antimicrobial action as the average bacterial concentration between 30-120h was 2.1 log_10_ CFU/mL lower in ATM + CAZ/AVI + IMI than in ATM + CAZ/AVI (2.44 log_10_ CFU/mL vs. 4.54 log_10_ CFU/mL, respectively). Adding IMI to ATM+ CAZ/AVI also resulted in an enhanced post-antibiotic effect. Although ATM + IMI reduced bacterial counts by ≥4 log_10_ CFU/mL in 6h time kill assays, regrowth to the system carrying capacity of ∼8.5 log_10_ CFU/mL by 24h in all arms rendered these drugs ineffective. By 2h of treatment, ATM alone resulted in long filamentous cells whereas IMI resulted in significant populations of spheroplasts. ATM+IMI caused blebbing in the center of elongating filaments followed by a burst in spheroplast formation. qRT-PCR showed upregulation of spheroplast protein Y (*spy)* and penicillin binding proteins (*pbp1, pbp2, pbp3*) for all combinations involving IMI. However, *pbp1, pbp3 and spy* remained unchanged and *pbp2* levels were downregulated by >25% in ATM monotherapy.

**Conclusion:**

Overall, this PDR clinical isolate persisted in the presence of ATM + CAZ/AVI. The addition of IMI to target PBP2 in β-lactam combinations maximally suppressed the emergence of filamentous persisters and may be a promising strategy to prepare for increasing rates of gram-negative resistance.

**Disclosures:**

**All Authors**: No reported disclosures

